# Comprehensive comparative study of Chiari-like malformation in veterinary and human medicine

**DOI:** 10.17221/125/2023-VETMED

**Published:** 2024-07-29

**Authors:** Jae-Hwan Jung, Hwi Park, Woo-Suk Kim, Hun-Young Yoon

**Affiliations:** ^1^Department of Veterinary Surgery, College of Veterinary Medicine, Konkuk University, Seoul, Republic of Korea; ^2^Department of Veterinary Internal Medicine, College of Veterinary Medicine, Konkuk University, Seoul, Republic of Korea; ^3^Department of Anatomy, College of Veterinary Medicine, and Veterinary Science Research Institute, Konkuk University, Seoul, Republic of Korea; ^4^KU Center for Animal Blood Medical Science, Konkuk University, Seoul, Republic of Korea

**Keywords:** Chiari-like malformation, Chiari type 1 malformation, cranioplasty, duraplasty, foramen magnum decompression, syringomyelia

## Abstract

This review aims to enrich our understanding of Chiari-like malformation (CLM) by combining human and veterinary insights, and providing a detailed cross-species overview. CLM is a developmental abnormality characterised by caudal displacement of the hindbrain into the foramen magnum due to an entire brain parenchymal shift caused by insufficient skull volume. This malformation leads to a progressive obstruction at the craniocervical junction, which disrupts the normal cerebrospinal fluid flow, leading to secondary syringomyelia. The clinical signs of CLM and syringomyelia include phantom scratching, head tilt, head tremor, ataxia, tetraparesis, pain, muscle atrophy, and scoliosis or torticollis. Magnetic resonance imaging remains the gold standard for diagnosing CLM, since it allows the visualisation of abnormal findings such as the caudal cerebellar herniation, caudal cerebellar compression from occipital dysplasia, and attenuated cerebrospinal fluid cisternae. Although various medical and surgical interventions, including foramen magnum decompression, can provide temporary symptomatic/clinical sign relief, current literature shows a lack of sustained long-term efficacy. Therefore, additional research is needed to evaluate the long-term effects of existing treatment strategies and to compare different techniques utilised in conjunction with foramen magnum decompression.

## INTRODUCTION

In humans, Chiari type 1 malformation, the most common type of Chiari malformation (CM), encompasses a variety of rhombencephalic anomalies characterised by caudal displacement of the cerebellar tonsils or vermis into the cervical spinal canal. This condition can lead to various abnormalities including syringomyelia, hydromyelia, hydrocephalus, kyphosis, and scoliosis (Azahraa Haddad et al. 2018). Meanwhile, Chiari-like malformation (CLM) is a developmental abnormality frequently observed in small and toy breed dogs, such as Pomeranian, Pug, Brussels Griffon, Chihuahua, Maltese, and Yorkshire terriers. However, the most frequently reported breed affected by this malformation is the Cavalier King Charles Spaniel (CKCS) (Dewey et al. 2005; Hood 2016). CLM is mostly reported in dogs but it has also been rarely observed in other species such as brachycephalic cats and lions (McCain et al. 2008; Korff and Willamson 2020). The estimated prevalence of CLM in CKCS is remarkably high, ranging between 92% and 100%, with syringomyelia (SM) diagnosed in up to 70% of the breed (Hood 2016). Complex malformation of the skull causes overcrowding of the brain parenchyma, resulting in cerebellar compression and subsequent herniation at the foramen magnum (Rusbridge 2020). Moreover, overcrowding of the craniocervical junction causes secondary obstruction of cerebrospinal fluid (CSF) flow, resulting in the formation of fluid-containing cavities in the parenchyma of the spinal cord (Rusbridge 2007). Based on the pathophysiological insights into CLM/SM, CLM in dogs serves as a model of naturally occurring human Chiari type 1 malformations and is reported to show similarities between species (Driver et al. 2013). Neuropathic pain (NP) emerges as the predominant clinical symptom of CLM and SM (Hood 2016).

The precise aetiology of CM is unknown, and while significant research efforts in human medicine are underway to unravel mechanisms underlying this intricate neurological disorder, little progress has been made. In veterinary medicine, there is also a wealth of research on CLM in dogs, but the exact aetiology remains unclear. This ongoing uncertainty in both fields contributes to a knowledge gap between the two fields. Therefore, this review aims to bridge the gap between veterinary and human medicine by providing a comprehensive analysis of CLM and SM. By addressing current limitations in the literature and highlighting the potential benefits of comparative studies, our goal is to facilitate a more cohesive and comprehensive understanding of CLM, to benefit both the veterinary and human medical understanding.

## PATHOGENESIS

In dogs, the occurrence of CLM is similar to that of Chiari malformation type 1 (CM1) in humans, where a reduced volume of the caudal fossa causes overcrowding of the brain parenchyma. This condition leads to the caudal displacement of the cerebellum and, in some cases, the brainstem into the foramen magnum (Hechler and Moore 2018). Ultimately, it can lead to cerebellar compression and herniation, alongside medullary kinking, occlusion of the dorsal craniocervical subarachnoid space, ventriculomegaly/hydrocephalus, turbulent CSF flow, and SM.

The role of morphological factors, particularly the hypoplasia of the basioccipital bone, has been extensively studied in human CM1. Similarly, research in dogs has explored skull morphometry to elucidate the aetiology of CLM/SM (Loughin 2016). Comparative analysis between CKCS and mesaticephalic breeds has revealed a shallower caudal cranial fossa (CCF) and abnormal suboccipital and basioccipital bones in CKCS, which leads to incongruity between the brain and cranial cavity (Loughin 2016). In CKCS with CLM, the spheno-occipital synchondrosis closes prematurely at 8 months, compared to 12 and 16 months in other brachycephalic and mesaticephalic breeds, respectively (Loughin 2016; Hechler and Moore 2018), resulting in an abnormally short and wide skull shape (Hechler and Moore 2018).

## CHIARI MALFORMATIONS IN HUMANS

CM in humans encompasses morphological and anatomical malformations of the posterior fossa and hindbrain, including the cerebellum, pons, and medulla oblongata (Hidalgo et al. 2017). It was originally classified as 4 distinct subtypes, discernible through imaging examination (Hidalgo et al. 2017). The most common form, CM1, is characterised by a protrusion of the cerebellar tonsils > 5 mm below the foramen magnum (Hidalgo et al. 2017). This herniation obstructs the cisterna magna and disrupts the juncture between the intracranial and intraspinal compartments, affecting the bidirectional flow of CSF and its pulse pressure equilibration (Shao et al. 2022). Such disruptions not only distort the viscoelastic support of the cerebellar tonsils and spinal cord, but also causes damage and dysfunction of craniospinal suspension structures, including extradural ligaments such as the myodural bridges, intradural dentate ligaments, and the arachnoid framework (Shao et al. 2022). Chiari malformation type 2 is characterised by the descent of the brainstem along with cerebellar tonsils, or vermis into the cervical spinal canal. It is less common and typically associated with an open distal spinal dysraphism, such as meningomyelocele (Hidalgo et al. 2017). Chiari malformation type 3 entails the herniation of the cerebellum into a high cervical or low occipital meningoencephalocele, with or without the brainstem (Hidalgo et al. 2017). Chiari type 4 involves a severe form of cerebellar hypoplasia or aplasia and tentorial hypoplasia, distinguished by the absence of herniation, similar to primary cerebellar agenesis (Hidalgo et al. 2017). However, this classification is now considered obsolete.

Chiari malformation types 0, 1.5, and 5 were not originally described by Hans Chiari and are not yet universally accepted terms (Hidalgo et al. 2017). Chiari type 0 initially represents SM involving a McRae line of less than 3 mm without apparent hindbrain herniation, but it has recently been used to present typical symptoms of CM1 with minimal cerebellar tonsilar herniation, whether or not SM is present (Hidalgo et al. 2017; Bogdanov et al. 2021; Medicis et al. 2023). Chiari malformation type 1.5 represents a more advanced stage of CM1, involving the descent of the cerebellar tonsils and brainstem, and could be associated with skeletal deformities of the craniocervical junction and cervical spine (Hidalgo et al. 2017; Giallongo et al. 2021; Vetrano et al. 2024). Finally, Chiari type 5, the most severe type, involves cerebellar agenesis along with the descent and herniation of the occipital lobe into the foramen magnum (Hidalgo et al. 2017; Noureldine et al. 2019).

## PREVALENCE OF CHIARI MALFORMATIONS

The incidence of CM in humans is rare, with a prevalence between 0.24–3.6% in the population. However, the actual incidence is estimated to be much higher, since many cases with the typical structural abnormalities of Chiari do not show clinical symptoms and thus remain undiagnosed (Holly and Batzdorf 2019). This condition is predominantly found in females, and because the cerebellar tonsils tend to slightly ascend with age, its prevalence is higher in the paediatric population than in adults (Holly and Batzdorf 2019). The peak occurrences of CM are reported in two age groups: children aged 8–9 years, and adults aged 41–46 years (Holly and Batzdorf 2019). Although the cause of these incidence peaks is unknown, variations in the therapies employed for infant and adult patients, and their respective outcomes, have been documented (Holly and Batzdorf 2019).

## SYRINGOMYELIA PATHOPHYSIOLOGY

Overcrowding of the caudal/posterior cranial fossa may lead to the formation of a syrinx which is a fluid-filled cystic region within the spinal cord parenchyma that is not lined by ependyma (Hood 2016; Loughin 2016). The initial expansion of the central canal of the spinal cord causes hydromyelia, where the cavity is lined with ependyma. As the disease progresses, the ependymal lining splits, allowing fluid to enter the grey matter of the spinal cord, leading to syringohydromyelia, which is only partially lined by ependyma (Hood 2016). Obstruction to CSF flow may also causes a syrinx form without continuity to the central canal. The term SM is generally used for all clinical conditions characterised by the spinal cord cavitation that involves fluid identical to or very similar to CSF as MR imaging cannot determine if a fluid cavity within the spinal cord is lined by ependyma (Rusbridge et al. 2006). As the syrinx expands, it may damage the spinothalamic tract fibres, causing pain and paraesthesia (Hood 2016). Further enlargement damages the ventral horn cells, leading to a reduction in spinal reflexes, muscle atrophy, and weakness. Additionally, the syrinx can extend along the entire length of the spinal cord (Hood 2016). Some theories propose that it is caused by a combination of reduced CSF absorption through nasal lymphatics, inadequate venous drainage, intracranial hypertension, altered neuroparenchymal compliance, and the conformation features of the spinal canal (Rusbridge and Knowler 2021). The mechanism underlying syrinx development secondary to CLM has not been fully elucidated and is considered multifactorial (Loughin 2016). The currently accepted paradigm for syrinx formation secondary to CM/CLM posits that obstruction to CSF flow in the subarachnoid space results in a timing discrepancy between the spinal arterial pulse peak pressure and the CSF pulse peak pressure (Rusbridge 2020). When the CSF pulse pressure peaks prematurely, the CSF flows into the perivascular space (Rusbridge 2020). This perivascular space fluctuates in size with the cardiac cycle, and it is the widest when spinal arteriolar pressure is low (Rusbridge 2020). When the peak CSF pressure is high, the perivascular space behaves similarly to a faulty one-way valve, allowing fluid to flow from the perivascular space into the central canal, thereby contributing to the formation of the syrinx (Rusbridge 2020). The occurrence of SM is associated with blockages occurring anywhere along the CSF pathway (Rusbridge and Knowler 2021). This phenomenon has been reported in various diseases including intracranial masses/cysts, spinal arachnoid diverticula, and inflammatory conditions; however, CLMs are the most common cause of SM in the veterinary field (MacKillop et al. 2006; Rusbridge and Knowler 2021).

In humans, primary spinal SM is strictly associated with intraspinal pathologies, such as arachnoid cysts of the spine, spinal trauma, or scarring from meningitis or subarachnoid haemorrhage (Holly and Batzdorf 2019). Notably, it occurs far less frequently than SM associated with CM (Holly and Batzdorf 2019).

Moreover, in dogs diagnosed with both CLM and SM, the anatomical abnormalities are generally more severe than in those with only CLM. These include pronounced brachycephaly, deformations of the craniocervical junction such as cervical flexure, kinking or elevation, and altered angulation at the craniospinal junction. Other changes comprise modifications in the angle of the axial dens, increased proximity of the atlas to the skull, which refers to atlanto-occipital overlap, the loss of the cisterna magna, and morphological changes affecting both the spinal canal and cord (Rusbridge and Knowler 2021).

## CLINICAL SIGNS IN DOGS WITH CHIARI-LIKE MALFORMATION/SYRINGOMYELIA

In dogs with CLM/SM, NP and related abnormal sensations tend to be the most common symptoms (Loughin 2016). Clinical manifestations of pain in dogs with CLMs are divided into those without clinical signs (CLM-N) and those with clinical signs of NP (CLM-P) (Rusbridge 2020). CLM-P, which can occur with or without syringomyelia, generally presents in a nonspecific manner (Rusbridge 2020). Dogs with CLM-P exhibit more pronounced brachycephaly, a reduced cranial base, and an increased degree of craniofacial hypoplasia, with greater neuroparenchymal disproportion and crowding (Rusbridge 2020). CLM-P is also associated with difficulties in equilibrating intracranial pressure, typically due to CSF pathway obstructions (Rusbridge 2020). CLM-P signs include head and ear scratching or rubbing, spinal pain, vocalisation, aversion to touch, exercise intolerance/reduced activity, sleep disruption, and behavioural changes such as increased anxiety or changes in social interactions (Rusbridge 2020).

Clinical signs of SM correlate with the size of syringes, wherein in dogs, particularly in the CKCS, a syrinx exceeding 4 mm in a maximum transverse width is considered large (Rusbridge 2020). Dogs with SM experience pain secondary to the destruction of the fibres in the dorsal horn laminae. The length and asymmetry of the syrinxes, as well as the maximum syrinx width, serve as strong predictors of pain (Loughin 2016). While cervical spinal pain is the main clinical manifestation of SM, additional clinical signs such as phantom scratching, scoliosis, cervicothoracic torticollis, weakness, thoracic limb muscle atrophy, and decreased postural responses are also reported (Rusbridge et al. 2006; Rusbridge 2020). Apart from phantom scratching, which involves a rhythmic scratching motion directed toward the neck without actual contact with the skin, the clinical presentation of SM is typically non-specific.

Therefore, other possible diagnoses to consider include intervertebral disk disease, stenosis, and infectious or inflammatory conditions such as meningitis (Rusbridge 2020). Neurological impairments associated with SM align with the location of the syrinx (Rusbridge 2020). The occurrence of phantom scratching corresponds to the existence of a sizable mid-cervical syrinx that extends to the superficial dorsal horn in the C3–C6 spinal cord segments (C2–C5 vertebrae) on the same side as the site of scratching (Rusbridge et al. 2019; Rusbridge 2020). Weakness of the paraspinal and thoracic limb muscles is associated with the presence of a large cervicothoracic syrinx, whereas thoracic limb muscle atrophy correlates with a large syrinx occupying the C5–T1 spinal segment (Rusbridge 2020). Scoliosis arises from damage to the lower motor neurons situated in the ventral grey matter of the spinal cord, which leads to paraspinal muscle atrophy and an asymmetric muscle tone (Loughin 2016).

## CLINICAL SYMPTOMS IN HUMANS WITH CHIARI MALFORMATION/SYRINGOMYELIA

The clinical presentation of CM1 in humans is generally similar between adults and paediatric patients; however, there are some differences (McCludgage and Oakes 2019). Pain, particularly in the craniocervical region, caused by Valsalva manoeuvres, represents the most common symptom in both age demographics (McCludgage and Oakes 2019). However, in nonverbal groups, such as infants and young paediatric patients, it is challenging to assess the headache symptom. Localised pain and symptoms often manifest later in the clinical course, after neurological sequelae have already occurred (McCludgage and Oakes 2019). Consequently, manifestations of brainstem dysfunction, such as central sleep apnoea or feeding difficulties, are more likely to occur (McCludgage and Oakes 2019). The symptoms commonly observed in CM1 can be grouped into three categories: those related to CSF obstruction, brainstem or cerebellar compression or dysfunction (including cranial nerves), and spinal cord dysfunction or SM (McCludgage and Oakes 2019). Valsalva- or strain-induced occipital or upper cervical pain/headaches are commonly associated with CM1. These are caused by the caudal displacement of the cerebellar tonsils or arachnoid occlusions within the CSF pathways, leading to obstruction and a consequent transient elevation in intracranial pressure (McCludgage and Oakes 2019). Moreover, reduced intracranial compliance has been implicated in hydrocephalus in humans with CM (McCludgage and Oakes 2019). The representative symptoms associated with brainstem compression, cerebellar compression, or cranial nerve dysfunction include a weak or absent gag reflex, hoarseness, and swallowing difficulties, predominantly from glossopharyngeal and vagus nerve impairment (McCludgage and Oakes 2019). Trigeminal, facial, abducens, and hypoglossal nerves may also be involved (McCludgage and Oakes 2019). Compression caused by herniated tonsils in CM1 can lead to direct neural damage, particularly due to the vulnerability of trigeminal nucleus fibres in the spinal tract, which have low myelin and are dorsally located (Novegno 2019). The prevalence of trigeminal neuralgia among CM1 cases is approximately 5–10% (Novegno 2019). Regarding facial neuropathy, factors such as direct compression on the nerve, abnormal CSF flow dynamics occurring at the craniocervical junction, or micro-ischaemic damage owing to the contact with vascular structures may be involved (Novegno 2019).

Brainstem or medullary compression can lead to central sleep apnoea and, more rarely, to autonomic symptoms such as sinus bradycardia, syncope, and drop attacks (McCludgage and Oakes 2019). Vestibular signs such as downbeat nystagmus, truncal ataxia, vertigo, dizziness, and tinnitus are associated with cervicomedullary junction dysfunction (McCludgage and Oakes 2019). Spinal cord dysfunction is closely related to SM associated with CM1 (McCludgage and Oakes 2019). Symptoms can include upper and lower motor neuron signs, sensory loss of pain and temperature sensation, spasticity, and weakness (McCludgage and Oakes 2019). In addition, scoliosis may be a sign of SM in the paediatric population, and it usually presents as thoracic levoscoliosis as opposed to the dextroscoliosis typical of idiopathic scoliosis (McCludgage and Oakes 2019). Some argue that the common occurrence of levoscoliosis in CM1 patients is related to herniation of the right cerebellar tonsil, and levoscoliosis reportedly appears in 90% of patients when the right tonsil is more caudally displaced than the left tonsil (Tubbs et al. 2002).

## DIAGNOSIS

Although computed tomography (CT) can better demonstrate structural changes in the bone, high-field magnetic resonance imaging (MRI) is considered the gold standard for diagnosing CM/CLM and SM (Hechler and Moore 2018). Linear and volume changes of the brain and cervical regions can be assessed using T1-weighted (T1 W) and T2-weighted (T2 W) sagittal and transverse images (Loughin 2016; Hechler and Moore 2018). In dogs, a diagnosis of CLM is made when MRI reveals caudal cerebellar compression, cerebellar vermis herniation, and attenuation of the CSF due to overcrowding of the caudal fossa, T2 W sagittal images tend to be the most useful sequence/plane for identifying CLM in dogs (Hood 2016; Loughin 2016). Often, CLM is accompanied by findings of SM, ventricular dilation/hydrocephalus, and medullary kinking (Loughin 2016). In humans, CM1 has clearer diagnostic criteria than in dogs, determined using MRI and the measurement of the McRae Line (Hidalgo et al. 2017). When measured with the McRae Line, a line drawn from the basion to the opisthion, the diagnosis is confirmed if one or both cerebellar tonsils protrude 5 mm or more below the foramen magnum (Hidalgo et al. 2017). On sagittal images, SM is identified as a linear T2 hyperintensity within the spinal cord, that typically extends across multiple vertebrae but can be focal (Loughin 2016). T2 W transverse images are used to assess the diameter of SM and the involvement of the dorsal horn (Loughin 2016). The MRI scan range should span from the interthalamic adhesion to C5. Given that syringes often extend into the thoracic spinal cord, some authors recommend imaging at least to the T3 (Hechler and Moore 2018). Syringes are most commonly located in the cervical and upper thoracic spinal cord often sparing the C1 spinal cord segment but may extend into the lumbar spinal cord. CLM will rarely cause cavitation/syrinx formation within the brainstem termed syringobulbia (Hechler and Moore 2018). According to one study, SM most frequently appears in the C1-4 and T2–L2 regions (Loughin 2016). In humans, differential diagnoses should consider other neurological conditions that cause similar symptoms or occur with CM, including craniocervical malformations, subarachnoid diverticulae, intracranial cysts, hydrocephalus, intervertebral disc disease, vertebral column malformations, and primary secretory otitis media (Hood 2016; Loughin 2016).

Morphological changes of CLM and SM can be easily confirmed using MR imaging; however, no specific imaging parameters consistently predict clinical sign severity or disease progression (Hechler and Moore 2018). Given the weak correlation between morphology and symptom/clinical sign severity, cine MRI or phase-contrast MRI techniques have been proposed to quantify CSF flow velocity and turbulence at the craniocervical junction in both veterinary and human medicine (Loughin 2016; Hechler and Moore 2018). This method combines flow-dependent contrast with cardiac gating, an MR sequence obtained from electrocardiogram recordings during image acquisition, allowing image generation throughout the cardiac cycle, including systole and diastole (Loughin 2016). This dynamic imaging technique provides information regarding CSF flow alterations across the cardiac cycle and detects increased cerebellar pulsation during systole (Loughin 2016). This technique demonstrates that flow obstruction at the foramen magnum causes turbulent flow correlating with the presence and severity of SM. Additionally, CSF flow evaluation is used to examine the effectiveness of decompressive surgery and monitor clinical progression (Loughin 2016).

In human medicine, diffusion tensor imaging has recently been employed to evaluate white matter tract dysfunction in the medullae of patients with CM1 (McCludgage and Oakes 2019). Patients with CM1 exhibit increased fractional anisotropy, compared to those without CM1, indicating a potential white matter tract dysfunction (McCludgage and Oakes 2019).

CT can be useful for the morphometric analysis of the cranial cavity and the CCF in canines diagnosed with CLM, as well as identifying concurrent craniocervical junction abnormalities, which may influence surgical interventions and long-term outcomes of the condition (Loughin 2016; Hechler and Moore 2018). It is imperative that CSF sampling and analysis are conducted during the thorough examination of patients with suspected CLM/SM to exclude inflammatory neurological diseases that present with clinical signs and MRI findings similar to those of SM (Hechler and Moore 2018).

The CSF analysis in dogs with CLM/SM may show high nucleated cell count (reference range, < 5 cells/μl), or increased protein levels (reference range, < 48 mg/dl lumbar and < 25 mg/dl cisterna magna), yet these values may also be normal (Loughin 2016). In one study analysing the CSF of dogs with CLM and SM, three of five dogs had normal results. One other dog showed a normal WBC count with a high protein concentration (79 mg/dl), albuminocytologic dissociation, and the other dog showed an increase in WBC count (10 WBC/μl) with a normal protein concentration (Dewey et al. 2005).

The British Veterinary Association (BVA) has developed CLM/SM grading guidelines to categorise the severity of structural abnormalities (Hechler and Moore 2018). The diagnosis of CLM is based on midsagittal T2 W images, with the severity of cerebellar prolapse significantly increasing when the head is flexed; thus, precise positioning during imaging is essential (Hechler and Moore 2018). To achieve accurate diagnostics, the dog’s head and neck should be extended so that the base of the skull aligns with the floor of the vertebral canal from C1 to C2 (BVA 2019).

The grading of CLM emphasises the configuration of the cerebellum and subarachnoid space over the morphology of the supraoccipital bone (Hechler and Moore 2018). The BVA characterises CLM into three grades for dogs: Grade 0 (normal), Grade 1 (mild CLM), and Grade 2 (substantial CLM) (BVA 2019). Grade 0 signifies a cerebellum with a rounded shape with a signal consistent with the CSF between the caudal cerebellar vermis and the foramen magnum (BVA 2019). Grade 1 depicts a cerebellum that lacks a rounded shape, displaying indentation by the supraoccipital bone, yet it maintains a CSF signal between the caudal vermis and the foramen magnum (BVA 2019). Grade 2 is characterised by a cerebellar vermis either impacted or herniated through the foramen magnum (BVA 2019). Syringes are diagnosed using standard T1 W or T2 W MRI sequences. Typically, syringes exhibit hypointensity on T1 W images and hyperintensity on T2 W images (BVA 2019). Pre-syrinx conditions, which are characterised by spinal cord oedema, hyperintensity on T2 W images and isointensity or slight hypointensity on T1 W images (BVA 2019). Similar to the CLM grading scheme, which is based on the BVA grading scheme, the SM grading scheme is determined by measuring the syringes’ maximum internal diameter on the transverse plane (BVA 2019). The SM classification includes three grades in canines: Grade 0 (normal), Grade 1 (mild SM), and Grade 2 (substantial SM) (BVA 2019). Grade 0 denotes the absence of central canal dilation or syrinx formation (BVA 2019). Grade 1 corresponds to a central canal dilation with an internal diameter of less than 2 mm (BVA 2019). Grade 2 is characterised by an SM with a central canal dilation diameter of ≥ 2 mm or the presence of a separate syrinx or pre-syrinx, regardless of central canal dilation (BVA 2019). Additionally, the grading system incorporates a letter designation reflecting the dog’s age group at the time of imaging (BVA 2019). Dogs older than 5 years receive a subtype “a,” those between 3 to 5 years are given subtype “b,” and those aged from 1 to 3 years are classified into subtype “c” (BVA 2019). Breeding recommendations exist according to SM’s grading scheme (BVA 2019). The purpose of these breeding guidelines is to exclude dogs with early-onset SM (BVA 2019). For SM grade 0a, the dog can be bred to any dog (BVA 2019). For SM grade 0b, the dog can be bred to dogs with SM grades 0a, 0b, 0c, and 1a (BVA 2019). For SM grade 0c, the dog can be bred to dogs with SM grades 0a, 0b, and 1a (BVA 2019). In case of SM grade 1a, the dog can be bred to any dog (BVA 2019). For SM grade 1b, 1c, 2a, and 2b, the dog can be bred to dogs of SM grades 0a and 1a (BVA 2019). It is not recommended to breed dogs with SM grade 2c or any dog with clinical signs of CLM/SM (BVA 2019).

When interpreting MR images scans, it is crucial to be aware of flow-related artifacts. In large breed dogs, pulsatile venous flow within the internal vertebral venous plexus can cause linear, vertical hyperintensities in the cranial to mid-cervical spinal cord on T2-weighted images (Seiler et al. 2011). These artifacts become more noticeable in transverse T1-weighted images, particularly before and after contrast administration, and are accompanied by typical ghosting seen in pulsatility artifacts (Seiler et al. 2011). To prevent misdiagnosing these artifacts as intramedullary lesions, the phase and frequency encoding direction may be switched or flow compensation and gradient moment nulling may be implemented to resolve the artifact (Seiler et al. 2011).

## TREATMENT

The treatment options for CLM/SM encompass medical and surgical approaches (Hood 2016). However, because the pathophysiology of CLM/SM has not been fully elucidated, a standardised treatment approach has not yet been established (Hood 2016).

Multimodal drug therapy is commonly employed as a medical intervention, initially alleviating the clinical signs of pain (Hood 2016). However, it often requires dose escalation due to an inadequate response (Hood 2016). Consequently, surgical intervention is advised to address the underlying structural abnormalities that are the primary cause of CLM/SM. While surgical outcomes are favourable, additional data on long-term follow-up is needed (Hood 2016).

## MEDICAL MANAGEMENT

The medical treatment of CLM or CM with SM aims to manage NP in dogs and humans (Hechler and Moore 2018; Barpujari 2023). Medical treatments include 1) analgesic drugs, 2) drugs that reduce CSF production, and 3) glucocorticoids (Park et al. 2017; Barpujari et al. 2023).

In particular, the latest trend in medical therapy in human medicine is aimed at preventing the long-term use of opioid analgesics for pain management (Barpujari et al. 2023). This is associated with adverse effects of opioid use such as vomiting, nausea, reduced gastrointestinal motility, urinary retention, increased tolerance and dependence, oversedation, and increased intracranial pressure (Barpujari et al. 2023). Using non-opioid agents, efforts are made to safely and sustainably manage pain in neurosurgical patients (Barpujari et al. 2023). Recently, this has been achieved by applying local anaesthetics and nerve blocks using liposomal bupivacaine or 4% lidocaine along with multimodal drug therapy, ensuring a safer and more effective management of CM pain (Barpujari et al. 2023).

1. Analgesic drugs include nonsteroidal anti-inflammatory drugs (NSAIDs), anticonvulsants (e.g., gabapentin and pregabalin), opioids, tricyclic antidepressants (TCAs), and *N*-methyl-d-aspartate (NMDA) receptor antagonists (Loughin 2016; Hechler and Moore 2018).
**NSAIDs**
  Spinal cord and peripheral nerve injuries lead to increased synthesis of cyclooxygenase (COX)-1 and COX-2, catalysing prostaglandin production which may play a role in the pathophysiology of NP (Hechler and Moore 2018; Barpujari et al. 2023). NSAIDs such as carprofen and meloxicam act by inhibiting COX enzymes, thereby reducing prostaglandin synthesis (Hechler and Moore 2018). Evidence supporting the efficacy of COX inhibiting NSAIDs in treating chronic NP in veterinary practice is limited (Hechler and Moore 2018). Despite being commonly prescribed, their ability to improve the quality of life in CKCS has not been adequately demonstrated (Hechler and Moore 2018). However, some studies suggest they may be more effective when used alongside anticonvulsant drugs (Corral 2021).
**Anticonvulsants**
  Since SM-associated pain is typically neuropathic, antiepileptic drugs such as gabapentin, pregabalin, and topiramate are often utilised in pain management (Corral 2021; Barpujari et al. 2023). Gabapentin and pregabalin are believed to relieve pain by binding to the upregulated voltage-gated calcium channels in the dorsal horn’s superficial laminae (Hechler and Moore 2018). They are also known to inhibit phantom scratching by targeting GABAergic inhibitory interneurons in the dorsal horn that regulate itch and proprioceptive transmission (Hechler and Moore 2018). Pregabalin may be considered in cases where gabapentin at high doses is ineffective (Hechler and Moore 2018). Topiramate’s mechanism of action is believed to involve the modulation of voltage-gated sodium and calcium channels, enhancement of GABA-activated chloride channels, and inhibition of excitatory glutamate receptors (Hechler and Moore 2018). A recent prospective, randomised, cross-over study has reported that the combined administration of carprofen with gabapentin or topiramate is more effective in improving the quality of life in CKCS with CLM/SM compared to the administration of carprofen alone (Plessas et al. 2015).
**Opioids**
  In human medicine, opioids are used as broad-spectrum analgesics effective for intense pain management (Smith 2012; Barpujari et al. 2023). While not universally effective for all types of chronic NP, they do provide relief for some patients (Smith 2012). Opioids are less effective against NP compared to nociceptive pain and their use is controversial owing to their habit-forming potential in humans (Smith 2012). Opioids demonstrate the highest efficacy in alleviating peripheral NP, followed by spinal NP, whereas supraspinal NP tends to be the least responsive (Smith 2012). In veterinary medicine, tramadol is used to manage NP in dogs with CLM/SM due to its action as a weak mu-receptor agonist and as a serotonin and norepinephrine reuptake inhibitor (Hechler and Moore 2018). Nevertheless, most veterinary research on tramadol focuses on acute nociceptive pain, and there is limited clinical evidence supporting its application for treating chronic NP; therefore, its use in CLM/SM is not recommended (Hechler and Moore 2018).
**Tricyclic antidepressants**
  Tricyclic antidepressants (TCA) are considered first-line therapies for NP management in humans but are rarely used in veterinary medicine for this purpose (Hechler and Moore 2018). Amitriptyline, a TCA, is a representative drug used in these cases (Hechler and Moore 2018). TCA work by antagonising voltage-gated sodium channels, glutamate receptors, and NMDA receptors, although the precise mechanism of NP relief is not fully understood (Sindrup et al. 2005; Hechler and Moore 2018). Their main effect is thought to be the regulation of noradrenaline and serotonin, which are the neurotransmitters involved in pain transmission, by inhibiting their presynaptic reuptake (Sindrup et al. 2005; Hechler and Moore 2018).
**NMDA receptor antagonists**
  Amantadine, an NMDA receptor antagonist, is used for the treatment of neuropathic and osteoarthritic pain in humans and dogs (Lascelles et al. 2008; Hechler and Moore 2018; Barpujari et al. 2023). NMDA receptors are involved in allodynia and the pain response to non-noxious stimuli (Hechler and Moore 2018). Chronic pain conditions can activate normally inactive NMDA receptors. Amantadine is known to maintain NMDA receptors in a closed position during prolonged depolarisation, thereby managing chronic pain associated with allodynia (Hechler and Moore 2018). It has been reported to decrease opioid tolerance, reduce central sensitisation, and enhance the analgesic effects of NSAIDs, gabapentin, and opioids (Hechler and Moore 2018). In veterinary medicine, there is a lack of evidence proving its efficacy in the treatment of NP but its potential to reduce nociception and improve the emotional components of pain suggests it might be beneficial in combination with other analgesics for severe cases of CLM/SM (Corral 2021).
2. Drugs that decrease CSF production include omeprazole, cimetidine, and acetazolamide (Hechler and Moore 2018; Rusbridge 2020).
**Omeprazole**
  Omeprazole, a proton pump inhibitor, has been known to reduce CSF production by inhibiting the activity of Na^+^/K^+^-ATPase in the choroid plexus (Hechler and Moore 2018). This reduction in CSF production leads to decreased CSF pressure and it may slow the progression of syringes, making omeprazole a recommended treatment option for dogs exhibiting symptoms of CSF accumulation, such as hydrocephalus and SM (Girod et al. 2016; Hechler and Moore 2018). One study reported a 26–50% reduction in CSF production after intravenous or ventriculocisternal administration of omeprazole in dogs (Hechler and Moore 2018); nevertheless, it is unclear whether a similar decrease in CSF production occurs after chronic or oral administration of the drug (Hechler and Moore 2018; Girod er al. 2019).
**Cimetidine**
  Cimetidine, an H2 receptor antagonist, decreases CSF secretion (Rusbridge 2013; Fernandes et al. 2019; Peih-Yik et al. 2020). Histamine H2 receptors are found in the parietal and choroid plexus cells, which regulate cyclic adenosine monophosphate, a compound crucial for CSF secretion (Rusbridge 2013; Fernandes et al. 2019; Peih-Yik et al. 2020). Therefore, cimetidine has been included in medical protocols for SM management to reduce CSF secretions (Fernandes et al. 2019).
**Acetazolamide**
  Acetazolamide, a carbonic anhydrase inhibitor, reduces CSF secretion by preventing the conversion of water and carbon dioxide into bicarbonate and hydrogen ions, respectively (Costello et al. 2019; Barpujari et al. 2023). It is commonly used to treat idiopathic intracranial hypertension in humans and has been reported to reduce intracranial pressure in animals by reducing CSF secretion (Jamshidi et al. 2022). In human medical studies, the resolution of CM using acetazolamide has been reported, and pharmacological treatment with acetazolamide is strongly recommended before proceeding with posterior fossa decompression (Barpujari et al. 2023).
3. Corticosteroids include prednisone, prednisolone, dexamethasone, and methylprednisolone (Hechler and Moore 2018; Rusbridge 2020).
**Corticosteroids**
  Corticosteroids inhibit the synthesis of COX-2 and phospholipase A2, thereby preventing the release of pro-inflammatory mediators and reducing levels of substance P (Hechler and Moore 2018). They are advantageous in treating CLM/SM due to their ability to reduce CSF production among other effects (Hechler and Moore 2018). Chronic corticosteroid administration may be necessary for some dogs with CLM/SM to control clinical signs. It is recommended to initiate treatment with an anti-inflammatory dose and gradually decrease it to the lowest effective dose over several weeks (Hechler and Moore 2018).

## SURGICAL MANAGEMENT

### Surgical management in dogs

Surgical treatment is recommended if side effects of drug treatment are observed or when neurological clinical symptoms persist without improvement despite drug treatment (Hechler and Moore 2018). In general, dogs with CLM-induced pain reactions are more likely to have a clearer indication for surgery and a better postoperative prognosis (Rusbridge 2007; Rusbridge 2020). In such cases, foramen magnum decompression is a surgical option (Rusbridge 2020). When SM clinical symptoms, such as phantom scratching or weakness, appear, there is a high possibility that these symptoms will persist or recur after surgery. This is because the syrinx remains even after surgical procedures, such as FMD (Rusbridge 2020).

Previously, there were no widely accepted standardised criteria for the surgical treatment of dogs with CLM/SM (Ortinau et al. 2015). The Mississippi State University College of Veterinary Medicine Neurosurgery proposed criteria for selecting surgical candidates (Ortinau et al. 2015). According to these criteria, a dog is considered a surgical candidate when there is no improvement in clinical symptoms after 2 weeks of medical treatment before surgery, and if it met the following three criteria: 1) MRI evidence of CLM and cervical SM, 2) Syrinx in the cervical spinal cord measuring ≥ 3 mm in diameter on transverse T2 MRI, 3) Clinical signs of phantom scratching, cervical pain or hypersensitivity, or thoracic limb paresis with no MRI/CSF evidence of other pathologies that could produce similar clinical signs (Ortinau et al. 2015).

In veterinary medicine, surgical options such as foramen magnum decompression (craniocervical decompression), ventriculoperitoneal shunting, and syringopleural or subarachnoid shunting are available for CLM/SM treatment. However, these do not address the underlying factors that cause SM (Rusbridge 2020). Consequently, the syrinx usually persists postoperatively, and no surgical method has proven effective in consistently resolving clinical symptoms accompanied by pain (Rusbridge 2020).

1. Foramen magnum decompression (craniocervical decompression) surgery
  Foramen magnum decompression (FMD) (craniocervical decompression) is a surgical procedure for decompressing the craniospinal junction by removing the supraoccipital bone and C1 cranial lamina and is in the most common procedure for dogs with CLM/SM (Rusbridge 2007; Rusbridge 2020).
  FMD involves the removal of the supraoccipital bone until the cerebellar vermis is adequately exposed (Rusbridge 2020). However, even if FMD is successfully performed, symptoms may recur or worsen as compressive scar tissue forms at the surgery site, which may occur as early as 2 months post-surgery (Loughin 2016; Rusbridge 2020). The recurrence of clinical signs is reported to be between 25 and 47% (Loughin 2016). In humans, reconstructive measures of the caudal occipital area post-surgery have been undertaken to reduce the development of scar tissue (Loughin 2016). Similarly, the veterinary field employs cranioplasty utilising FMD in combination with a titanium mesh or polymethyl-methacrylate plate (Loughin 2016) (Table 1). In addition to FMD combined with titanium-mesh cranioplasty, the approach to this surgery varies, including techniques such as FMD with durotomy, FMD with both cranioplasty and durotomy, as well as FMD with durotomy followed by duraplasty (Ortinau et al. 2015).
  Dural opening, a treatment option for FMD, can involve durotomy and duraplasty (Rusbridge et al. 2006).
  While some surgeons assert that the act of opening the dural itself may increase the risk of complications, others contend that durotomy allows the identification and removal of other factors obstructing CSF flow, such as focal scar, dural prolapse, and intradural adhesion (Rusbridge et al. 2006; Pijpker et al. 2019). While this has not been extensively studied, many neurosurgeons routinely prefer dural opening in humans and favour this method (Rusbridge et al. 2006).
  Although these various surgical methods are often deemed successful in terms of immediate postoperative pain relief, there is a lack of data concerning long-term outcomes, and there are no specific criteria for the selective application of these surgical techniques (Ortinau et al. 2015). Moreover, even if surgery is successful, long-term drug management is required in most cases (Rusbridge 2020).
  Consequently, clinical improvements after FMD surgery are largely attributable to the decompressive effect of CSF pathways. This indicates that while FMD is not a direct treatment for SM, it is appropriate for patients with pain caused by CLM (Rusbridge 2020).
2. Shunting procedures
  Syringo-subarachnoid (S-S), syringo-peritoneal, or syringo-pleural shunting is the process of placing a shunt within the syrinx to enable fluid drainage to the subarachnoid space, peritoneal cavity, or pleural cavity (Koyanagi and Houkin 2010; Cucos et al. 2018; Rusbridge 2020). In human cases of CM/SM, shunting directly into a cavity has been shown to collapse the syrinx in the short term (Rusbridge 2007).
  However, on long-term follow-up, the results were poor due to shunt obstruction and/or spinal cord tethering, leading to a general aversion to this method in human medicine (Rusbridge 2007). Similarly, in veterinary medicine, the procedure is not commonly used in dogs. It is typically reserved for cases where a syrinx persists or progresses even after foramen magnum decompression or other surgical options are deemed unsuitable (Rusbridge et al. 2006; Rusbridge 2020).
3. Ventricular to peritoneal shunting
  Ventriculoperitoneal shunting is commonly used in human patients with hydrocephalus, where CSF is diverted from the ventricles to the peritoneal cavity (Kitagawa et al. 2008). This procedure can also be performed in dogs and cats with greatly enlarged ventricles or hydrocephalus and has been shown to cause syrinx regression (Rusbridge 2020). Ventriculoperitoneal shunting has been reported to have an incidence of complications of up to 25% including infection, blockage, disconnection, kinking of the catheter system, subdural haematoma, and fluid accumulation (Kitagawa et al. 2008; Rusbridge 2020).

### Surgical management in humans

In human medicine, surgical intervention for CM with an associated syrinx is generally limited to patients with overt symptoms. Accepted surgical indications for both paediatric and adult patients with CM1, are as follows: 1) individuals experiencing typical Valsalva-induced headaches; 2) those presenting with a concurrent syrinx; and 3) patients exhibiting neurological complications linked to abnormalities at the foramen magnum, the cervicomedullary junction, or dysfunction of the lower cranial nerves (McCludgage and Oakes 2019).

Surgical treatment in both humans and dogs aims to alleviate brainstem compression and distortion of cranial nerves, ensure adequate CSF flow within the cranial compartment, and reduce the syrinx cavity when present (Holly and Batzdorf 2019). Some neurosurgeons opt to reduce the size of the cerebellar tonsils, with the understanding that the CSF pulsations in an expanded cisterna magna contribute to the elevation and propagation of the tonsils into the spinal subarachnoid space (Holly and Batzdorf 2019).

Surgical approaches to CMs differ between children and adults, with posterior fossa decompression in children being less extensive (Holly and Batzdorf 2019). While the reason for this distinction is unclear, it may be due to the unique physical properties of a child’s tissue, including dural and neural tissues (Holly and Batzdorf 2019). It has been reported that subocciptal craniectomy alone is sufficient for children, and when treating confirmed tonsillar descent, it should be managed considering the potential for future elevation (Holly and Batzdorf 2019).

In human and veterinary medicine, neurosurgeons lack a consensus on the optimal surgical approach for CM1 (Pijpker et al. 2019). Therefore various surgical methods are applied based on specialist preference, ranging from posterior fossa decompression (PFD) alone to PFD with durotomy, duraplasty, and cranioplasty (Pijpker et al. 2019). Recently, the most performed procedures involve occipital bone removal to expose the cerebellar tonsils, atlas laminectomy, intradural exploration to identify obstructive membranes, manipulation of the tonsils with or without reduction, and duraplasty (Holly and Batzdorf 2019) (Table 1). In addition, human medicine tends to prefer minimally invasive surgical methods (Pijpker et al. 2019).

**Table 1:** Representative additional surgical methods performed after occipital bone decompression in veterinary and human medicine

Additional surgical methods following occipital bone decompression	Purposes (advantages)	Complications (disadvantages)
Duraplasty (with/ or without arachnoid preservation)	Augments the effect of bony decompression, especially if arachnoid and dura mater become scarred or hypertrophied (Dewey et al. 2005; Hwang et al. 2023). Enlarges subarachnoid space at the level of craniovertebral junction (CVJ) (Ozlen et al. 2021). Optimise expansion of cisterna magna to maintain fullness of subarachnoid space and ensure smooth flow of cerebrospinal fluid (Holly and Batzdorf 2019; Lou et al. 2019). Maximises expansion of the posterior cranial fossa (Hwang et al. 2023). Identifies and removes obstruction factors of CSF flow, allowing CSF to flow smoothly (Rusbridge et al. 2006; Lou et al. 2019). Maximises the syrinx-volume reduction rate (Holly and Batzdorf 2019; Hwang et al. 2023). Reduces scoliosis curve progression (Sadler et al. 2021).	If haemorrhage enters the subarachnoid space through the opening of the spinal dura and is poorly absorbed, fever and head and neck pain may occur (Lou et al. 2019). Contamination of the subarachnoid space due to meningeal vessel bleeding (Holly and Batzdorf 2019)*.* Increased rates of CSF leakage-related complications based on longer dura incision, including CSF leakage into peripheral tissues, subcutaneous exudate, pseudomeningocele, meningoencephalitis, and intracranial infection (Dewey et al. 2005; Lou et al. 2019; Osborne-Grinter et al. 2021). Increased risk of arachnoid scarring hyperplasia and muscle contractures, which compress the posterior cranial fossa, reducing effectiveness (Lou et al. 2019; Pijpker et al. 2019). Dural grafts pressed against the cerebellum by the cervical syringohydromyelia fluid creating a constrictive effect at the cervicomedullary junction (Dewey et al. 2005). If fascia is used for dural grafting, this devascularised tissue may cause adhesions or form a scar at the cervicomedullary junction, resulting in worse outcomes than the original constriction. (Deweu et al. 2005).
Cranioplasty (occipital bone reconstruction)	Enables extensive decompression to expand the area around the foramen magnum and the entire posterior cranial fossa (along the sigmoid sinus and transverse sinus) (Nishikawa et al. 2022). Normalises the volume of posterior cranial fossa (VPCF) and major cisterns suitable for decompression of the cerebellum and brainstem, and enables the remodelling of the proper positional relationship between the cerebellum, brainstem and occipital bone (Nishikawa et al. 2022). Protects the enlarged cisterna magna from pressure and adhesions of nuchal musculature and from extradural scarring (Pijpker et al. 2019). Reduces frequently reported complications (CSF leakage, pseudomeningoceles, extradural scarring at the surgical site) after PFD (Pijpker et al. 2019). Improves long-term outcomes following surgery (Pijpker et al. 2019).	No standard procedure for reconstructing the posterior cranial fossa, and little is known about the best technique (Pijpker et al. 2019). Due to the lack of consensus on optimal craniectomy size, the size of bone decompression and implant is based on experience rather than quantitative clinical evidence (Pijpker et al. 2019). Improperly designed implant castings, such as PMMA, may be placed in an unintended and inappropriate manner (Pijpker et al. 2019). Operations are longer, and cost effectiveness is reduced (Pijpker et al. 2019).

The size of the craniectomy opening for PFD varies among surgeons (Holly and Batzdorf 2019). Sufficient occipital bone should be removed to expose the cerebellar tonsils (Holly and Batzdorf 2019). However, an excessively wide craniectomy increases the risk of inducing cerebellar herniation into the spinal canal, compression on the brainstem, formation of arachnoidal scarring, obstruction of the CSF circulation, and stretching of a dural graft (Caffo et al. 2019). The size of the craniectomy opening is generally measured as 20–25 mm in width and 15–25 mm in vertical distance from the foramen magnum, based on measurements obtained from patient imaging (Holly and Batzdorf 2019; McCludgage and Oakes 2019). Generally, only a C1 laminectomy is performed, leaving the C2 lamina intact, and the atlantooccipital membrane is resected (Holly and Batzdorf 2019). The dorsal arch and muscular attachments of C2 must be preserved to prevent postoperative cervical swan neck deformity, which can cause subluxation and kyphosis, usually accompanied by compensatory lordosis (McCludgage and Oakes 2019). Since the pathology is confined to the foramen magnum and the area below the C1 arch, bone decompression should also be confined to this area, avoiding more extensive bone removal (McCludgage and Oakes 2019).

After completing bone decompression, the decision to open the dura must be carefully considered (McCludgage and Oakes 2019). It is important to consider the risk of missing potential complications from a dural opening and the unresolved pathology of the fourth ventricle, which is not relieved by bony decompression alone if dural opening is not performed (McCludgage and Oakes 2019). According to a specific report, 12% of individuals diagnosed with SM had an arachnoid veil observed at the outlet of the fourth ventricle (McCludgage and Oakes 2019). The dura is incised in a Y-shaped manner to optimise effective decompression and subsequent expansion of the cisterna magna (Holly and Batzdorf 2019). The arachnoid can then be opened, and the adhesions around the tonsils are broken down, which allows the tonsils to move (Holly and Batzdorf 2019). Care should be taken to avoid visualising and damaging the posterior inferior cerebellar arteries (Holly and Batzdorf 2019).

Since dogs do not have cerebellar tonsils and the cerebellar vermis is herniated, resection of the vermis is not performed following craniectomy because serious complications such as postoperative dysmetria, ataxia, and intention tremors are expected to occur (Rusbridge 2007). Conversely, in humans, the cerebellar tonsils herniate into the foramen magnum rather than the cerebellar vermis (Rusbridge 2007). Therefore resection or bipolar coagulation of the cerebellar tonsils can be performed to open the fourth ventricular outlet (Holly and Batzdorf 2019; McCludgage and Oakes 2019). Although tonsillar resection using a subpial technique is rarely performed in situations where bipolar electrocautery does not respond adequately or the tonsils are bulky, this technique requires great care (Holly and Batzdorf 2019; McCludgage and Oakes 2019). Additionally, it is essential to inspect the fourth ventricular outlet for scarring or an arachnoid veil and to dissect and open it if identified to ensure proper CSF flow (McCludgage and Oakes 2019).

Preserving the arachnoid layer without opening it may result in missed intradural pathology, whereas opening it can introduce blood into the subarachnoid space, increasing the risk of CSF leakage and postoperative scarring (Holly and Batzdorf 2019). Some sources advocate that arachnoid preservation may offer a safer and more effective approach by reducing complication risks while preserving the quality of PFD (Osborne-Grinter et al. 2021).

PFD with duraplasty using materials such as allografts, autologous tissue, and synthetic materials has shown a greater reduction in the syrinx cavity compared to bone decompression alone (Holly and Batzdorf 2019). Posterior fossa reconstruction has been reported to result in fewer postoperative complications, especially in the paediatric population, with a lower incidence of CSF leakage and pseudomeningocele compared to PFD alone (Pijpker et al. 2019). Rigid cranioplasty following PFD can protect the dilated cisterna magna by reducing nuchal muscle pressure and extradural scarring (Pijpker et al. 2019).

However, to date no standard procedure for posterior fossa reconstruction has been established (Pijpker et al. 2019). Titanium plates, polymethylmethacrylate (PMMA) bone cement, autologous cranial bone, and autologous iliac bone have been selectively used in various reconstructive techniques (Pijpker et al. 2019). Recently, it was demonstrated that by using a sonolucent cranial implant, point-of-care imaging can be provided long after the implant is applied (Mehta et al. 2023). Sonolucent PMMA-based implants permit transcranioplasty ultrasonography, providing clear images of the intracranial anatomy and pathology, and enhancing the follow-up monitoring in various neurosurgical contexts, including treatments for CM and ventriculoperitoneal shunt for hydrocephalus (Mehta et al. 2023).

## POSTOPERATIVE PROGNOSIS IN DOGS AND HUMANS

Although the short-term surgical success rate for FMD surgery in dogs with CLM/SM is approximately 80%, 25–47% of cases treated in this manner have been reported to recur and worsen over time (Rusbridge 2007; Fossum et al. 2019). The predominant cause for the recurrence or worsening of symptoms is believed to be the development of compressive or constrictive scar tissue (Loughin 2016). Due to the progressive nature of CLM/SM in dogs, a long-term follow-up of at least 2 years is necessary to evaluate the success of the surgical management (Rusbridge 2007).

In humans, additional surgeries are required in about 8–30% of cases following CM1 surgery (Dewey et al. 2005). Several CM 1 relapses have been reported to result from arachnoid scarring, intradural adhesions, and dural prolapse, which can lead to restenosis of the cisterna magna and obstruction of CSF flow, ultimately negating the benefits of PFD surgery and prompting the return of CM1 symptoms (Pijpker et al. 2019).

Approximately 80% of dogs with CLM and syrinx show improvement, such as some pain relief after surgery, but the syrinx is typically not resolved (Rusbridge 2007). This contrasts with the results in human medicine, where it is noted that clinical signs of syrinx are improved or fully resolved in more than 90% of patients with CM after cranio-cervical decompression, irrespective of the surgical technique applied (Rusbridge 2007). Several factors may account for the difference in syrinx resolution between dogs and humans following surgery (Rusbridge 2007).

Surgery performed in dogs does not sufficiently improve CSF flow through the foramen magnum, suggesting that removing most of the supraoccipital bone and dorsal laminae of C1 along with durotomy or cranioplasty is insufficient (Rusbridge 2007). Anatomical distinctions between humans and dogs allow for a more extensive lateral bone removal from the occipital bone and atlas in humans (Rusbridge 2007). Although not routinely performed in humans, it is possible to secure the space of the cisterna magna by moving, resecting, or performing bipolar coagulation of the cerebellar tonsils (Rusbridge 2007).

## CONCLUSIONS

In dogs, CLM has been recognised to parallel the CM1 observed in humans, establishing a critical link between veterinary and human medicine (Hood 2016).

However, despite the cross-species relevance, the underlying disease pathophysiology remains unclear in both species, and there is a lack of consensus on a standardised treatment approach (Zisakis et al. 2023).

Current diagnostic and therapeutic strategies, focusing on alleviating clinical symptoms such as NP and phantom scratching, offer short-term relief but long-term outcomes reveal a concerning tendency of symptom/clinical sign recurrence (Hechler and Moore 2018; Rusbridge 2020). This persistent challenge underscores the need for a more comprehensive examination of CLM, transcending the boundaries of veterinary and human medicine.

This review addresses the existing gaps in understanding by providing a thorough comparison of CLM in veterinary and human contexts. Recognising commonalities in clinical presentations and treatment responses allows practitioners to tailor their approaches, addressing the unique needs of individual patients and improving long-term outcomes.

The parallels and distinctions identified in this study serve as a foundation for future research, offering the potential to deepen our understanding of shared pathophysiological mechanisms underlying CLM and CM. Future research efforts could focus on exploring molecular and genetic factors contributing to the development of CLM in both species, potentially guiding the development of targeted therapeutic interventions, including medical or surgical treatment.
